# Learning Covariate Relations in Disease Progression Models Using Symbolic Neural Networks

**DOI:** 10.1002/psp4.70214

**Published:** 2026-03-10

**Authors:** Jesper Sundell, Ylva Wahlquist, Maria C. Kjellsson, Mats O. Karlsson, Kristian Soltesz

**Affiliations:** ^1^ Department of Automatic Control Lund University Lund Sweden; ^2^ Department of Pharmacy Uppsala University Uppsala Sweden

**Keywords:** diabetes, machine learning, Markov models, neural networks, pharmacometrics

## Abstract

Covariate modeling provides individual predictions of outcomes by disease progression models. Current methodology for mapping covariates onto model parameters is limited by predefined parametric functions which can result in inadequate covariate selection and biased predictions by the final model. Furthermore, present methodology scales poorly to high‐dimensional data due to combinatorial limitations. In the present study, a novel method for automation of covariate model identification in disease progression models is described. Symbolic neural networks are used to simultaneously identify the parametric covariate functions and optimize model parameters of a Markov chain. By stepwise pruning of initially fully connected dense symbolic networks, humanly readable functions representing the covariate relations are produced. The presented methodology is applied to a dataset containing disease progression observations for type 2 diabetes patients. Although utilizing fewer covariates, the resulting model demonstrates predictive performance similar to that of a model which was developed on the same data using state‐of‐the‐art modeling methodology.

## Study Highlights

- What is the current knowledge on the topic?
  ○ Current covariate model development tools are limited by predefined parametric functions which can result in inadequate covariate selection and biased predictions by the final model. Furthermore, present methodology scales poorly to high‐dimensional data due to combinatorial limitations.
- What question did this study address?
  ○ This study describes a novel covariate modeling method for disease progression models, which do not rely on predefined parametric functions. The method is applied to a type 2 diabetes disease progression dataset.
- What does this study add to our knowledge?
  ○ Application of the method results in a model which displays similar predictive performance as a model which was developed on the same dataset using state‐of‐the‐art modeling practice although utilizing fewer covariates.
- How might this change drug discovery, development, and/or therapeutics?
  ○ The method provides automatic and less constrained identification of covariate models, ultimately providing disease progression models which may more accurately predict individual risks.

## 1 Introduction

Disease progression modeling, used to quantitatively describe the evolution of disease severity over time, is a fundamental component in the pharmacometric toolkit. Probabilistic models, such as time‐to‐event models, constitute a significant proportion of disease progression models for single outcomes of interest. If multiple outcomes are analyzed, Markov chains may be used to jointly model all outcomes. Such models are often referred to as multistate models and typically contain competing risks [1].

Markov chains are composed of a finite set of states and parameterized in transition probabilities or transition rates. Applied to disease progression, Markov chains describe the movement of patients between states and the population's state occupation probability evolving over time. The per time probability to transition between two states may be linked to patient specific features such as age, sex, and biomarkers, referred to as covariates. Identification of such a covariate‐parameter relation (i.e., covariate model) is a cornerstone in pharmacometrics.

Markov models are increasingly used to analyze event data with multiple outcomes of interest [2]. In parallel, the interest in using machine learning has recently been growing in the pharmacometric community [3]. Several approaches for covariate selection have been suggested for pharmacokinetic models as summarized in a recent review [4]. However, machine learning‐based methods for covariate modeling of Markov models have, to the best of our knowledge, been underexplored.

The aim of covariate modeling includes both identification of clinically relevant covariates and the functional form of their mapping onto model parameters. The resulting model allows for predictions of individual outcomes. Assessment of covariates in pharmacometric models is currently performed as an iterative, stepwise process where a set of parametric functions is evaluated [5, 6]. Although multiple covariates may be mapped to the same parameter, functions including covariate interactions are seldom tested unless supported by empirical knowledge [7]. Using predefined parametric functions, however, may introduce bias in individual predictions and limit identification of clinically relevant covariates. Furthermore, current stepwise covariate model development methods are restricted by dimensionality since datasets with numerous covariates create combinatorial challenges. To mitigate such limitations, we have previously proposed the use of symbolic neural networks (SNNs) [8].

Similarly to regular artificial neural networks, SNNs can approximate underlying functions describing data [9, 10]. However, in contrast to artificial neural networks, readable equations representing the functions can be obtained by pruning (i.e., reducing) the network if requested by the user. SNNs can therefore be used for automatic covariate selection and identification of the parametric function [8, 11].

In this study, we demonstrate the use of SNNs in a probabilistic disease progression model. We model type 2 diabetes disease progression data using a framework for discrete‐time neural Markov models [12]. The covariate model describing the relationship between covariates and transition probabilities is identified using SNNs. The final model is validated and compared to a previously described model (hereafter referred to as the Kunina model) which was developed on the same dataset using current state‐of‐the‐art modeling practice [13].

## 2 Methods

### 2.1 Data

In short, the dataset consisted of observations from a total of 41,517 adult patients with type 2 diabetes, available in the Swedish National Diabetes register between the years 2005 and 2013. All patients were newly diagnosed (≤ 1 year since diagnosis), had at least one measurement of relevant covariates (Table 1) and were observed over a time period, in which, they could experience events representative of disease progression including diabetic kidney disease (DKD), macrovascular complications (MVC), or death. Followup times varied from 0.01 to 9.86 years with a median of 3.22 years. Demographics of patients included in the analysis are summarized in Table 1. If the observation period ended prior to death, the patient measurement was considered subject to right censoring. Since the exact times of developing DKD or MVC were unknown, the majority of disease progression event times were subject to interval censoring. A detailed description of the dataset has been published elsewhere [13]. For details on how to access data and recreate the dataset, please contact M Kjellsson.

**TABLE 1:** Baseline demographics of type 2 diabetes patients included in the analysis.

Characteristic	Median (range)
Age (years)	62.0 (18.0–99.0)
Duration of diabetes (years)	3.1 (0.01–9.6)
Triglycerides (mmol/L)	1.7 (0.3–16.0)
BMI (kg/m^2^)	29.8 (14.2–50.0)
HbA1c (mmol/mol)	48.0 (26.0–144.0)
sBP (mmHg)	135 (80–250)
dBP (mmHg)	80 (40–130)
HDL (mmol/L)	1.2 (0.3–4.0)
LDL (mmol/L)	3.1 (0.4–8.7)
Sex (Men/Women)	22,715/18,802

Abbreviations: BMI, body mass index; dBP, diastolic blood pressure; HbA1c, hemoglobin A1c; HDL, high‐density lipoprotein; LDL, low‐density lipoprotein; sBP, systolic blood pressure.

The dataset had previously been divided into two fixed datasets: training data (N=31,077) and validation data (N=10,440, 25% of total data) [13]. The split was performed so that all transitions were represented in both the training data and the validation data. The same split was used in this study to avoid effects on comparison between the SNN model and the Kunina model. The validation data was excluded during the entire training and was only used to evaluate the final model. In this work, 10% of training data was used as test data (see Section 2.5). The proportions of different events were considered when splitting the training data so that the proportions of events in the test data accurately reflected the training data. Distributions of events and patient demographics for the different datasets are summarized in the Supporting Information.

### 2.2 Markov Model

**FIGURE 1:**
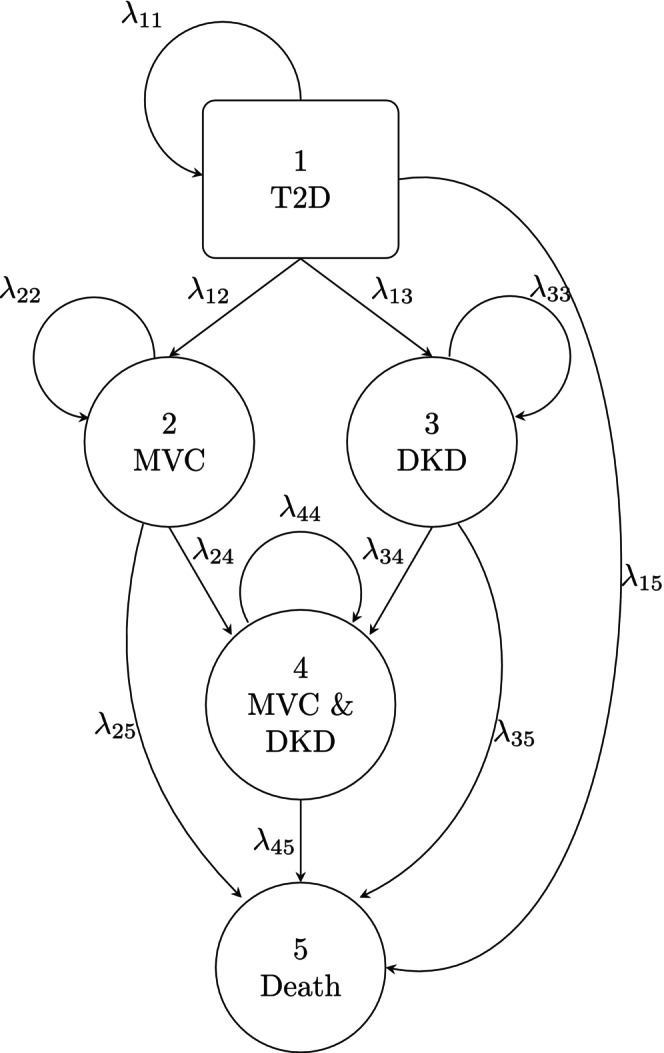
Illustration of the diabetes disease progression model. The probability to transition from state m to state n in each discrete time step is represented by $\lambda_{\mathrm{mn}}$. DKD, diabetic kidney disease; MVC, macrovascular complications; T2D, type 2 diabetes.

An equidistant discrete‐time Markov model with time steps of 1 month and states $s\in \left\{1,2,3,4,5\right\}$ was used to model the disease progression for type 2 diabetes patients. A graphical representation of the model is depicted in Figure 1. All patients started in state 1, representing type 2 diabetes. From there, patients could transit into either state 2 (MVC) or state 3 (DKD). From state 2 and state 3, patients could transit into state 4 equivalent of both MVC and DKD. Transitions into an absorbing state (state 5, death) were possible from all other states. All transitions were irreversible. The model can be represented by the transition probability matrix

(1) $$P_{\lambda }=\left\{\begin{array}{ccccc} \lambda_{11} & \lambda_{12} & \lambda_{13} & \lambda_{14} & \lambda_{15}\\ \lambda_{21} & \lambda_{22} & \lambda_{23} & \lambda_{24} & \lambda_{25}\\ \lambda_{31} & \lambda_{32} & \lambda_{33} & \lambda_{34} & \lambda_{35}\\ \lambda_{41} & \lambda_{42} & \lambda_{43} & \lambda_{44} & \lambda_{45}\\ \lambda_{51} & \lambda_{52} & \lambda_{53} & \lambda_{54} & \lambda_{55} \end{array}\right\}=\left\{\begin{array}{ccccc} \lambda_{11} & \lambda_{12} & \lambda_{13} & 0 & \lambda_{15}\\ 0 & \lambda_{22} & 0 & \lambda_{24} & \lambda_{25}\\ 0 & 0 & \lambda_{33} & \lambda_{34} & \lambda_{35}\\ 0 & 0 & 0 & \lambda_{44} & \lambda_{45}\\ 0 & 0 & 0 & 0 & 1 \end{array}\right\}$$

where $\lambda_{\mathrm{mn}}$ is the probability of transitioning from state = m to state = n. Here, the diagonal elements of the transition probability matrix are derived as $\lambda_{\mathrm{mm}}=1-\sum_{\begin{array}{c} \;n=1\\ \;n\neq m \end{array}}^{5}\left(\lambda_{\mathrm{mn}}\right)$. The initial probabilities of being in each state were $\pi_{0}=\left(1,0,0,0,0\right)$.

### 2.3 Covariate Model

In this work, the covariate model F is a function used to map covariate vector $\varphi ={\left[\varphi_{1},\varphi_{2}\ldots ,\varphi_{n_{\varphi }}\right]}^{⊤}$, of length $n_{\varphi }$, to $P_{\lambda }$. Thus, F can be represented by the matrix

(2) $$F=\left\{\begin{array}{c} f_{11}\ldots f_{1n}\\ \vdots \ddots \vdots \\ f_{m1}\ldots f_{\mathrm{mn}} \end{array}\right\}$$

where each element maps φ to a transition probability $\lambda_{\mathrm{mn}}$. To prevent scale‐based influence of covariates on model parameters, all continuous covariates were normalized between 0 and 1. The only binary categorical covariate, sex, was set to −0.5 and 0.5. Equivalently to the work performed by Kunina et al. [13], covariate values were fixed to baseline values with the exception of age which was allowed to increase over time. Since living with type 2 diabetes and related comorbidities affects the probability of death over time, time spent in state was used as covariate in addition to covariates in Table 1 for all transitions to state 5. By using equivalent covariate input to the model as Kunina et al. used in their model, potential differences in model performance related to covariate information was neglected.

### 2.4 Symbolic Neural Networks

**FIGURE 2:**
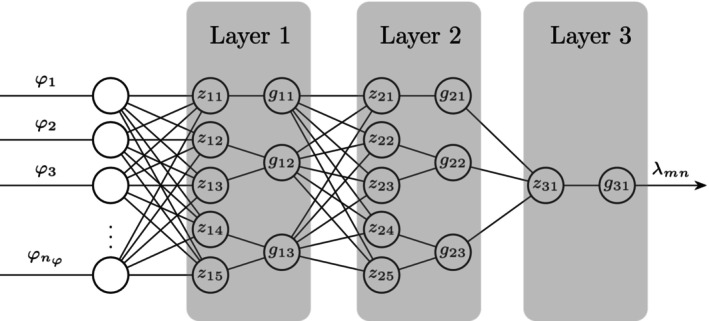
Representation of the symbolic neural networks with three layers used to map covariates φ to transition probabilities $\lambda_{\mathrm{mn}}$ between the *m*th and the *n*th state. $z_{ln_{z}}$ are inputs which are subject to affine transformation into the *l*th layer and $g_{ln_{g}}$ are the activation functions in the *l*th layer.

To characterize the relationship between covariates and transition probabilities, we used SNNs as mapping functions $f_{\mathrm{mn}}$. SNNs are small artificial neural networks where regular arithmetic operations constitute the activation functions. Since the activation functions differs between nodes and layers, pruning of the SNNs (as described below) can result in humanly readable expressions. The potential final expressions thus depend on the degree of pruning and the activation functions defined. The SNN structure used in this study is illustrated in Figure 2.

A neural network consists of $n_{l}$ layers where the output of each layer ($x_{l+1}$) is an affine transformation of the input ($x_{l}$) into layer l followed by application of a vector of nonlinear activation functions $g_{l}$:

(3) $$\begin{array}{c} z_{l}=W_{l}x_{l}+b_{l}\\ x_{l+1}=g_{l}\left(z_{l}\right). \end{array}$$

The weight matrices $W_{l}$ and bias vectors $b_{l}$ contain parameters which are optimized during model training. In this work, the vector of activation functions of length $z_{n}$ applied to the input $z_{l}={\left[z_{l1},z_{l2}\ldots z_{ln_{z}}\right]}^{⊤}$ for layer $l\in \left\{1,2,3\right\}$ were

(4) $$\begin{array}{c} g_{1}\left(z_{1}\right)=\left[\begin{array}{c} z_{11}\\ z_{12}\cdot z_{13}\\ {\left|z_{14}\right|}^{z_{15}} \end{array}\right],\\ g_{2}\left(z_{2}\right)=\left[\begin{array}{c} z_{21}\\ z_{22}\cdot z_{23}\\ \frac{z_{24}}{|z_{25}|+1} \end{array}\right],\\ g_{3}\left(z_{3}\right)=\sigma \left(z_{3}\right) \end{array}$$

where σ is the sigmoid function assuring that the SNN output $g_{3}\left(z_{3}\right)\in \left[0,1\right]$. The denominator in the division was used to constrain the output of $g_{2}$ within a finite range. The activation functions were chosen so that common mappings such as linear and power functions as well as more complex functions including covariate interactions could be identified by the pruning process. Although further complexity could be added by, for example, adding additional hidden layers to increase the number of potential output functions, the complexity chosen was considered adequate for this dataset.

### 2.5 Training

Training is referring to an iterative optimization of model parameters by minimization of a loss function. In this work, models were trained by minimization of the negative log‐likelihood described below.

We consider equidistant time steps k of 1 month. Observed transitions consisted of two types of observations. For a subset of observations K, the exact time of transition or right censoring was known to occur at time $t=k^{*}$. For a second subset I, transitions were known to occur in the interval $I^{*}=\left(k_{1}^{*},k_{2}^{*}\right)$, that is, interval censored observations. The joint log‐likelihood function L of λ conditional on both I and K for a time invariant transition probability was thus

(5) $$\begin{array}{c} L\left(\lambda |I,K\right)=\sum_{I^{*}\in I}\log\left(\sum_{k=k_{1}^{*}}^{k_{2}^{*}}\lambda {\left[1-\sum_{\begin{array}{c} n=1\\ n\neq m \end{array}}^{N}\lambda_{\mathrm{mn}}\right]}^{k}\right)+\\ \sum_{k^{*}\in K}\alpha \log\lambda +k^{*}\log\left(1-\sum_{\begin{array}{c} n=1\\ n\neq m \end{array}}^{N}\lambda_{\mathrm{mn}}\right) \end{array}$$

where

(6) $$\alpha =\left\{\begin{array}{ll} 1, & \text{if}\;a\;\text{transition occurred}\;\mathrm{at}\;t=k^{*}\\ 0, & \text{if the patient is subject to right censoring} \end{array}\right.$$

and *N* is the number of possible transitions from the *m*th state. To account for a time‐varying probabilities, the likelihood function was modified slightly to

(7) $$\begin{array}{c} L\left(\lambda |I,K,t\right)=\sum_{I^{*}\in I}\log\left(\sum_{k=k_{1}^{*}}^{k_{2}^{*}}\lambda \left(t\right)\prod_{k=0}^{k-1}\left[1-\sum_{\begin{array}{c} n=1\\ n\neq m \end{array}}^{N}\lambda_{\mathrm{mn}}\left(t\right)\right]\right)+\\ \sum_{k^{*}\in K}\alpha \log\lambda \left(t\right)+\prod_{k=0}^{k^{*}-1}\log\left(1-\sum_{\begin{array}{c} n=1\\ n\neq m \end{array}}^{N}\lambda_{\mathrm{mn}}\left(t\right)\right) \end{array}$$

thus allowing the transition probability to be different in each discrete time step. A full derivation of the likelihood function is provided in [12].

In each training iteration, the loss for the test data was evaluated. During the initial training, prior to the first pruning step, convergence was considered when the loss of the test data did not improve by more than 0.01 for 35 consecutive iterations. During pruning, the number of consecutive iterations without improvement in test data loss required to stop training was set to 25.

To prevent the sum of all transition probabilities in each row of the transition probability matrix to become higher than 1 during training, a boundary was included according to:

$$\begin{array}{l} \text{if}\;\sum_{n=1}^{N}\left(\lambda_{\mathrm{mn}}\right)\geq 1\;\text{then}\\ \;\lambda_{m1}=\lambda_{m1}\\ \;\lambda_{m2}=\lambda_{m2}\left(1-\lambda_{m1}\right)\\ \;\lambda_{\mathrm{mn}}=\lambda_{\mathrm{mn}}\left(1-\lambda_{m1}\right)\ldots \left(1-\lambda_{\mathrm{mn}-1}\right).\\ \mathrm{end}\;\text{if} \end{array}$$

The methodology was implemented in Julia language (version 1.11) using neural networks from the Lux package (version 1.12.4) [14]. The adaptive optimizer ADAM with a training rate of 0.001 was used for loss function minimization [15]. The implementation is disclosed in the GitHub repository [16].

### 2.6 Pruning

Network pruning was aiming to reduce the fully connected dense SNNs until sparse SNNs equivalent of readable equations remained. Pruning of the networks was performed by iteratively alternating between training the networks until convergence and removing parameters. In the first pruning iteration, 20 parameters were removed, followed by 10 in the second iteration and five parameters in the third and forth iterations. From the fifth iteration and onward, one parameter was removed per iteration. The number of parameters pruned in each iteration was arbitrarily selected. Pruning continued until any significant change in the loss value was observed. However, a maximum of 10 parameters per $f_{\mathrm{mn}}$ was allowed.

In each pruning step, the parameters of lowest importance were removed. Parameter importance was determined using both the loss sensitivity to perturbation of parameters and parameter magnitudes by calculation of parameter saliences S according to

(8) $$S\left(\gamma_{k}\right)=\gamma_{k}^{2}H_{k}$$

where $H_{k}$ is the *k*th element of the diagonal Hessian of the loss function with respect to parameter $\gamma_{k}$.

The time required to obtain H is disproportionally increasing as the number of observations increase. A sampling‐based approach was therefore used to approximate H for SNNs representing transition probabilities where most observations were available (i.e., transitions from state 1). The approximated diagonal Hessian elements $H_{k}^{\prime }$ were obtained by

(9) $$H_{k}^{\prime }=\text{median}\left(H_{\mathrm{kb}}\right)$$

where $H_{\mathrm{kb}}$ are diagonal Hessian elements derived using the *b*th batch of observations sampled from the dataset. Sampling was performed by taking ten samples from the dataset. Each sample included 30 observed transitions from each possible transition and 30 right censored observations. A comparison between H and $H^{\prime }$ is provided in the supporting information (Figures S1 and S2).

### 2.7 Model Evaluation

To visually evaluate model predictions, a visual predictive check (VPC) was performed for the validation dataset. The VPC was conducted by comparing nonparametric probabilities of state occupancy over time for the validation data to non‐parametric probabilities for datasets generated using the final trained and pruned model and the covariates from the validation data as input. Non‐parametric probabilities were derived using the Aalen‐Johansen estimator [17].

Right censoring was randomly added to generated datasets by replacing the last simulated time of event $k_{s_{i}}$ for each simulated individual with

(10) $$k_{s_{i}}^{*}=\min\left(k_{s_{i}},c_{s_{i}}\right)$$

where $c_{s_{i}}$ is a simulated time of censoring. If the censoring time occurred prior to the last simulated event time, all events following the time of censoring were removed. The simulated times of censoring were sampled from a mixture model containing an exponential component and a gamma component. Parameters of the mixture model were optimized by modeling the distribution of censoring times from the original dataset using the ExpectationMaximization package (version 0.2.4) in Julia.

Our model and the Kunina model were compared using the Brier score and Kullback–Leibler (KL) divergence at 1 year following type 2 diabetes diagnosis [18, 19]. Both metrics reflect predictive performance by the models where a lower value is equivalent to more accurate predictions.

The Brier score was computed using the predicted state occupation probabilities $\pi_{i}\left(t\right)$:

(11) $$\mathrm{BS}\left(t\right)=\frac{1}{N_{i}}\sum_{i=1}^{N_{i}}{\left(y_{i}\left(t\right)-\pi_{i}\left(t\right)\right)}^{2}.$$

where $y_{i}\left(t\right)=\left\{s_{1},s_{2}\ldots s_{5}\right\}$ is the observed state occupation vector for the *i*th individual and

(12) $$\begin{array}{c} y_{i,s}\left(t\right)=\\ \left\{\begin{array}{cc} 1, & \text{if vector element}\;s\;\text{is the state},\text{in which},\text{the patient resides}\\ 0, & \text{otherwise}. \end{array}\right. \end{array}$$

For our model, the individual state occupation probabilities were derived using the initial state occupation probabilities $\pi_{0}=\left(1,0,0,0,0\right)$ and the individual time‐varying transition probability matrices $P_{\mathrm{\lambda i}}\left(t\right)$ according to

(13) $$\pi_{i}\left(t\right)=\pi_{0}\prod_{t=0}^{T}P_{\mathrm{\lambda i}}\left(t\right).$$

Individual state occupation probabilities from the Kunina model were derived by simulations using the ordinary differential equations constituting the model and individual sets of parameters as described in [13]. The individual contribution to the KL divergence for each model was

(14) $${\mathrm{KL}}_{i}\left(P_{i}\|\pi_{i}\right)=\sum_{s=1}^{s=5}P_{i,s}\left(t\right)\log\frac{P_{i,s}\left(t\right)}{\pi_{i,s}\left(t\right)}.$$

Here, $P_{i,s}\left(t\right)$ are pseudo‐observations obtained by

(15) $$P_{i,s}\left(t\right)=N_{i}{\hat{P}}_{s}\left(t\right)-\left(N_{i}-1\right){\hat{P}}_{s}^{-i}\left(t\right)$$

where ${\hat{P}}_{s}\left(t\right)$ is the observed Aalen‐Johansen state occupation probability estimated on the whole dataset and ${\hat{P}}_{s}^{-i}\left(t\right)$ is the corresponding state occupation probability estimated on the dataset when the *i*th individual is excluded [17, 20]. Pseudo‐observations were used to account for right‐censored observations.

## 3 Results

The final covariate model, mapping covariates to transition probabilities of the type 2 diabetes disease progression model, included age and state occupation time as covariates according to:

(16a) $$f_{12}=\sigma \left(-7.35+3.34\mathrm{AG}E^{2}\right)$$

(16b) $$f_{13}=\sigma \left(-3.77-\frac{4.41}{{\left(1.61\mathrm{AGE}\right)}^{2.26\mathrm{AGE}}}\right)$$

(16c) $$f_{15}=\sigma \left(-5.1+2.93T+3.96\mathrm{AGE}-3.67\mathrm{AG}E^{-1.32\mathrm{AGE}}\right)$$

(16d) $$f_{24}=\sigma \left(-13.69+9.08\mathrm{AGE}\right)$$

(16e) $$f_{25}=\sigma \left(-11.64+5.87\mathrm{AGE}+5.56\cdot T\cdot \mathrm{AGE}\right)$$

(16f) $$f_{34}=\sigma \left(-5.88\right)$$

(16g) $$f_{35}=\sigma \left(\frac{-4.33}{{\left(0.34T+0.79\mathrm{AGE}\right)}^{1.98}+0.33}\right)$$

(16h) $$f_{45}=\sigma \left(-12.8+4.28T+8.47\mathrm{AGE}\right).$$

Probabilities to remain in the current state are derived as $\lambda_{11}=1-\left(f_{12}+f_{13}+f_{15}\right)$, $\lambda_{22}=1-\left(f_{24}+f_{25}\right)$, $\lambda_{33}=1-\left(f_{34}+f_{35}\right)$ and $\lambda_{44}=1-f_{45}$, respectively. Note that AGE is the age in years normalized by dividing with the highest age achieved in the training dataset (102.07 years) and T is the state occupancy time (in months) divided by the maximum time spent in the respective states. The maximum state occupation times for states 1, 2, 3 and 4 were 114, 95, 97 and 78 months, respectively. The final loss normalized by number of observations $\left(\text{loss}/N_{\mathrm{obs}}\right)$ for the training dataset, the test dataset and the validation dataset were similar (0.88, 0.91 and 0.9, respectively) indicating adequate generalization capabilities by the model.

**FIGURE 3:**
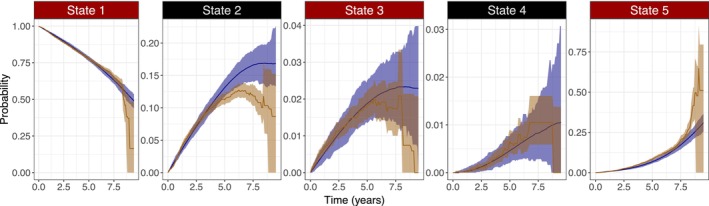
Visual predictive check for the validation data. Blue line and blue area is the median and range, respectively, of non‐parametric state occupation probabilities based on 1000 simulated datasets. Brown line is the non‐parametric state occupation probability for the validation data and brown area is the corresponding 95% confidence interval derived from a bootstrap ($N_{\text{bootstrap}}=1000$).

A VPC displaying the non‐parametric observed and model generated state occupation probabilities over time for the validation data is presented in Figure 3. A VPC excluding right censoring in the generated datasets is illustrated in the Supporting Information. Overall, the prediction interval and the confidence interval of the state occupation probabilities overlapped well. The mixture model used to model the distribution of censoring times is described in the Supporting Information.

**FIGURE 4:**
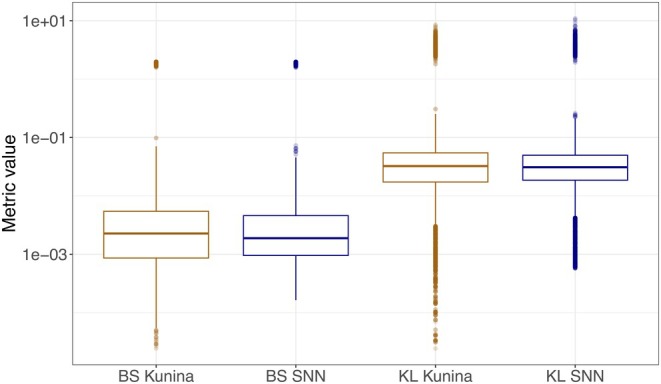
Comparison of Brier score (BS) and Kullback–Leibler divergence (KL) between the model produced by the symbolic neural networks (SNN) and the Kunina model based on the validation data.

The Kunina model included three covariates: age, state occupancy time, and sex, whereas the SNN model included only age and state occupancy time. Similarly to the Kunina model, age was allowed to increase over time in the SNN model, allowing for a fair comparison. Although the SNN model included fewer covariates than the Kunina model, the predictive performance of the SNN model was marginally better than that of the Kunina model. Different summary metrics of predictive performance based on both models applied to the validation data are presented in Table 2. The distributions of both the Brier score and the Kullback–Leibler divergence were similar for both models, as depicted in Figure 4.

**TABLE 2:** Summary of performance metrics for the symbolic neural network model and the Kunina model based on the validation data.

	SNN model	Kunina model
Kullback–Leibler divergence		
Mean	0.178	0.18
Mode	0.031	0.042
Median	0.031	0.032
Brier score		
Mean	0.094	0.094
Mode	0.0016	0.0028
Median	0.0019	0.0023

*Note:* A lower value is equivalent of better performance.

Abbreviation: SNN, symbolic neural network.

## 4 Discussion

We present a novel methodology for automated covariate model development for Markov models. Unlike commonly used covariate model building tools, such as the stepwise covariate modeling, which iteratively evaluates predefined covariate relations, our method smoothly shapes the final covariate‐parameter expressions. The SNN method simultaneously selects covariates and their mapping while forming the equation, thus exhausting the search space of parametric functions. Such an approach offers a major advantage over current methodology since less bias due to predefined functions is introduced. By reducing such a bias, the likelihood of identifying influential covariates increases. Furthermore, parametric functions which would not be evaluated using traditional approaches are tested, which promotes the discovery of novel covariate mappings.

In this work, the activation functions and architecture of the SNNs were designed so that common covariate relations such as a linear or a power relation could be identified. However, the design and choice of activation functions may be adapted based on any prior knowledge. The method thus, similarly to current practice, allows for expert knowledge to be inserted into the covariate model building process. Of note, the final expressions produced by the SNNs may vary depending on the architecture and activation functions specified in the networks. For optimal gain in using our method, the initial SNNs should therefore be adequately complex in their design. To determine if the specified SNNs are of such adequate complexity, the user may first apply a model containing regular artificial neural networks of sufficient size and depth to maximize predictions. If predictions by the fully dense SNN model and the model containing regular networks are similar, the complexity of the SNNs can be considered to be adequate.

The aim of the present methodology is to identify expressions mapping covariates to model parameters, which maximize individual predictions of probabilities. Given the initially large search space, the final expressions may vary depending on network initializations. Although such varying expressions are expected to predict probabilities similarly, further investigation of the effect of initialization and pruning strategies would be interesting to perform to gain knowledge of method consistency. Such an investigation may further increase the understanding of uncertainties in predictions.

Overfitting, which results in poor generalizability by the model, is a well known challenge in machine learning. To overcome overfitting, we continuously evaluated the model on test data during training. Training was stopped when model performance on the test data reached a plateau. By specifying a convergence criteria based on unseen test data, the training focused on improving predictions. Importantly, a major difference between regular neural networks and the algorithm presented herein is the pruning process which constitutes a natural regularization of overfitting. The combination of pruning and the convergence criteria used therefore maximizes the generalizability of the final model. Although not used in this work, additional evaluation of overfitting by the final model can be performed by using cross‐validation if desired by the user. Then, if the predictive performance differs significantly between the training data and test data, generalization properties can be tuned by, for example, performing a higher degree of pruning.

Our method is based on a discrete‐time framework in contrast to the Kunina model which was developed using continuous time. An advantage of using a continuous‐time framework is the predictability independent of specific time points. However, clinical data is discrete in nature and a per time probability, such as the monthly probability of disease progression used in this work, can be considered to be of adequate clinical relevance. Regarding predictions of type 2 diabetes disease progression, little would be gained from a more granular prediction. If more granular predictions would be of clinical interest, a higher resolution of the time variable may be used. However, increasing resolution also increases computational time and a balance between the two should be considered. Notably, since the models were developed in different temporal frameworks, comparison of the models was performed at a specific time point where predictions could be extracted from both models. A comparison at 1 year following type 2 diabetes diagnosis was used since the initiation of diabetes therapy will affect biomarkers and disease progression over time and information regarding diabetes therapy was excluded from the original analysis [13].

In this work, network pruning was performed solely based on parameter importance. The pruning strategy naturally results in removal of covariates. Although clinically relevant covariates could be removed during the pruning if the pruning is too severe, such removal would affect the loss value wherefore the loss value should be monitored. For datasets containing numerous covariates, a pruning strategy could include an initial step of covariate reduction. The importance of covariates can be quantified by evaluation of the Hessian of the loss function with respect to each covariate. Including a covariate reduction step would, however, require specifying an additional hyperparameter controlling the stopping criteria for covariate removal.

According to the performance metrics, the SNN model performed slightly better than the Kunina model, although the difference in predictive performance was negligible. The primary difference was that the Kunina model included sex as a covariate in addition to age and time since diagnosis. Sex is an established predictor of mortality [21]. However, no effect of sex on mortality was suggested by our framework. Interestingly, for some parameters in the Kunina model, the differences in parameter values between sexes were either low or the parameter precisions were poor, indicating that only minor or uncertain effects of sex on those parameters may be present.

The VPC suggested that the overall trend in model predictions corresponded to the data. However, as reflected by the illustration, uncertainties in predictions should be considered when predicting state occupation over time. The most notable deviation in state occupation probability between data and predictions was observed for state 2. The lack of data in the later phase contributes to such deviation. However, the initiation of therapy also decreases the overall risk of developing comorbidities which may contribute to the overprediction of occupying state 2.

Disease progression models can, through predictions, be used to guide medical interventions. By including covariate relations in the model, predictions, and thus interventions, can be performed on an individual level. Contemporary automatic covariate modeling methods focus on covariate screening to identify relevant covariates followed by manual adjustment and testing of a few predefined covariate relations. Our method automates the entire covariate modeling process while simultaneously exploring a substantially larger search space of covariate relations. Increasing the search space further increases the likelihood of identifying relevant covariates and may provide more accurate mappings of covariate relations, resulting in better individual predictions. The resulting model may, if supported by the data, thus yield a more accurate tool for clinicians should it be used to guide interventions.

Random effects are commonly included in pharmacometric models to account for uncertainty in individual predictions. Although Markov chains are probabilistic, random effects could be used to provide uncertainties in the probabilities. Employing our methodology in a mixed‐effects framework is possible. However, simultaneous estimation of random effects would be computationally demanding. Should the user want to include random effects, the covariate model can first be obtained using a SNN approach followed by the addition of random effects which can be estimated in a mixed‐effects framework [11].

In conclusion, a novel methodology for automated modeling of covariate relations in Markov chains is presented. The methodology offers advantages over contemporary methods since no assumptions regarding the functional form of the parametric functions are required. Such an increased flexibility in the covariate model can improve individual predictions, ultimately providing a more accurate tool to guide clinical interventions. By application on a dataset containing type 2 diabetes disease progression data, the method is demonstrated to perform similarly to current state‐of‐the‐art modeling practice with fewer covariates.

## Author Contributions

J.S. wrote the manuscript. All authors designed the research. J.S. performed the research. J.S. analyzed the data.

## Funding

This work was partially supported by the Wallenberg AI, Autonomous Systems and Software Program (WASP) funded by the Knut and Alice Wallenberg Foundation and a grant from the Knut and Alice Wallenberg Foundation to SciLifeLab for research in Data‐driven Life Science, DDLS (KAW 2020.0239). J.S., Y.W. and K.S. are members of the ELLIIT Strategic Research Area at Lund University.

## Conflicts of Interest

The authors declare no conflicts of interest.

## Supporting information


**Data S1:** psp470214‐sup‐0001‐DataS1.pdf.
