# Supplementary material for: Learning Covariate Relations in Disease Progression Models Using Symbolic Neural Networks
Source: CPT Pharmacometrics Syst Pharmacol. 2026 Mar 10;15(3):e70214. doi: 10.1002/psp4.70214 (PMC13014801; doi:10.1002/psp4.70214)
Supplement: Supplementary file 1 — Data S1: psp470214‐sup‐0001‐DataS1.pdf. [file PSP4-15-e70214-s001.pdf]

Supplement to Learning covariate relations in disease progression models using symbolic neural networks

1 Data preparation

The original Kunina dataset consisted of continuous time observations (in years). To fit observations into the discrete-time framework with each time step representing one month, event and censoring times were multiplied by twelve and then rounded to the closest integer. Transitions from state 1 to state 4 (n=62) were excluded during this analysis.

Only baseline covariate values were used in this work. For patients where covariate information was missing during the first observation, the first observed value was set to baseline. For patients where no covariate value had been registered, the median value for that covariate was used. The proportion of missing values for each covariate was $\leq 11.6\%$.

2 Data distributions

Distributions of data for the entire dataset and the divided dataset.

Table S1: Baseline demographics of type 2 diabetes patients included in the analysis presented as median (range) or counts.

Characteristic	Entire data	Training data	Test data	Validation data
Age (years)	62.0 (18.0-99.0)	62.0 (18.0-99.0)	62.0 (22.0-94.0)	62.0 (18.0-95.0)
Triglycerides (mmol/L)	1.7 (0.3-16.0)	1.6 (0.3-16.0)	1.6 (0.3-12.3)	1.7 (0.3-15.4)
BMI (kg/m^2)	29.8 (14.2-50.0)	29.8 (15.2-50.0)	29.8 (16.5-49.7)	29.8 (14.2-50.0)
HbA1c (mmol/mol)	48.0 (26.0-144.0)	48.0 (26.0-144.0)	48.0 (26.0-137.0)	48.0 (26.0-141.0)
sBP (mmHg)	135 (80-250)	135 (80-250)	135 (90-220)	135 (80-250)
dBP (mmHg)	80 (40-130)	80 (40-130)	80 (45-130)	80 (40-130)
HDL (mmol/L)	1.2 (0.3-4.0)	1.2 (0.3-4.0)	1.2 (0.4-4.0)	1.2 (0.3-4.0)
LDL (mmol/L)	3.1 (0.4-8.7)	3.1 (0.4-8.7)	3.1 (0.4-8.6)	3.1 (0.4-8.4)
Sex (Men/Women)	22,715/18,802	15,326/12,643	1686/1422	5703/4137

BMI, body mass index; HbA1c, hemoglobin A1c; sBP, systolic blood pressure; dBP, diastolic blood pressure; HDL, high-density lipoprotein; LDL, low-density lipoprotein.

Table S2: Distribution of events and censoring in the population and the datasets used for training and validation presented as counts (%).

Event	Entire data	Training data	Test data	Validation data
Censored state 1	34,970 (84.2)	23,539 (84.2)	2616 (84.2)	8815 (84.4)
12	3878 (9.3)	2656 (9.5)	280 (9.0)	942 (9.0)
13	660 (1.6)	442 (1.6)	50 (1.6)	168 (1.6)
15	1947 (4.7)	1297 (4.6)	155 (5.0)	495 (4.7)
Censored state 2	3434 (88.6)	2353 (89.0)	261 (89.1)	820 (87.0)
24	114 (2.9)	74 (2.8)	7 (2.4)	33 (3.5)
25	330 (8.5)	216 (8.2)	25 (8.5)	89 (9.4)
Censored state 3	493 (74.7)	326 (73.6)	36 (73.5)	131 (78.0)
34	54 (8.2)	38 (8.6)	8 (16.3)	8 (4.8)
35	113 (17.1)	79 (17.8)	5 (10.2)	29 (17.3)
Censored state 4	182 (79.1)	121 (79.6)	14 (82.4)	47 (77.0)
45	48 (20.9)	31 (20.4)	3 (17.6)	14 (23.0)

3 Parameter pruning

The time required to obtain parameter saliences is disproportional to the number of individuals assuming one observation per individual as illustrated in Figure S1. Parameter saliences for networks which were trained using the highest number of observations (i.e. networks representing probabilities of transitions from state 1) were therefore approximated using the median of diagonal hessians of the loss function with respect to the parameters based on subsets of the data. Approximated saliences versus saliences obtained using all observations from subset of the diabetes data set is depicted in Figure S2.

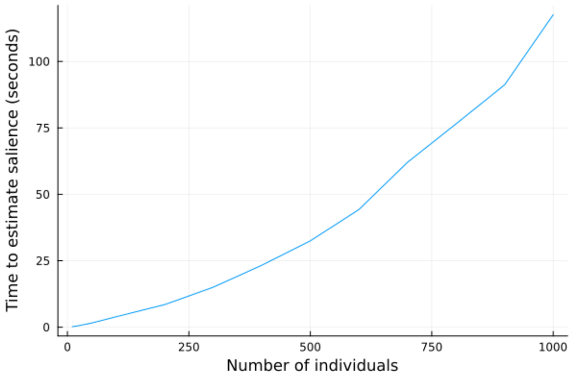

Figure S1: Number of individuals included in the analysis versus time required to estimate parameter salience.

Note that since we are using salience to remove parameters of low importance, the approximate rank of each salience value related to a parameter in relation to other salience values is of importance

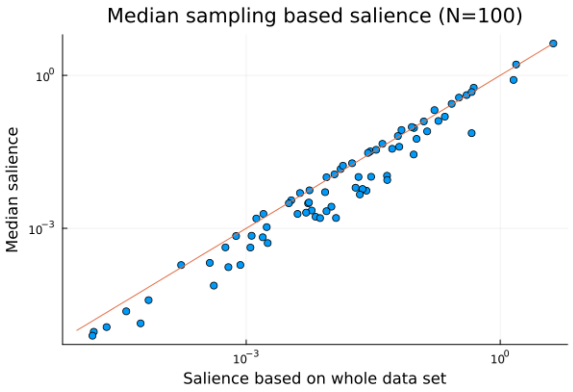

Figure S2: Salience calculated using all observations (N=1000) versus approximated (median) salience based on subsets of observations (N=100).

rather than the actual value of the salience. Further note, that if parameters are removed and the network is trained further, the relative order of parameter salience may change. Parameter saliences were therefore re-calculated in each pruning iteration.

4 Mixture model

The mixture model used to generate censoring times c_{s_i} for simulations consisted of an exponential component and a gamma component:

$$\mathcal{P}(c_{s_i}) = \phi f_{Exp}(c_{s_i}|\theta) + (1-\phi) f_{Gamma}(c_{s_i}|\alpha_g, \beta) \tag{1}$$

where ϕ is the mixture weight, θ is the rate parameter of the exponential distribution, and α_g and β are the shape and scale parameters of the gamma distribution, respectively. Optimized parameter values were: $\phi = 0.5$, $\theta = 21.1$, $\alpha_g = 9.0$ and $\beta = 6.4$. The distribution of censoring times and the probability density function of the mixture model are depicted in Figure S4.

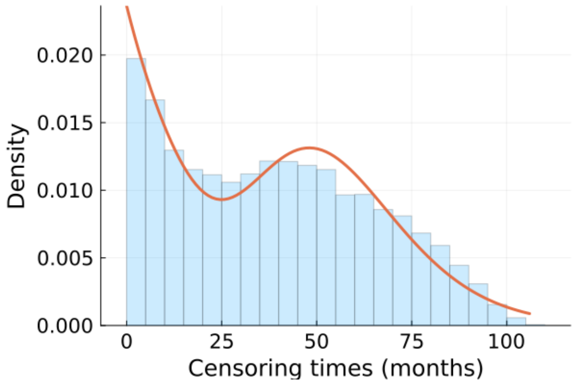

Figure S3: Distribution of censoring times. Red line is the probability density function for the mixture model.

5 VPC excluding right censoring in the generated datasets

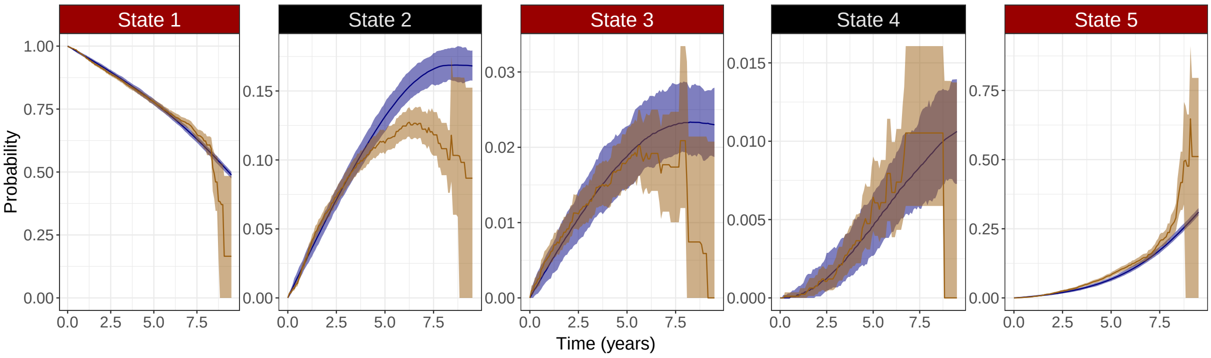

Figure S4: Visual predictive check for the validation data excluding rigth censoring in the generated datasets. Blue line and blue area is the median and range, respectively, of non-parametric state occupation probabilities based on 1000 simulated datasets. Brown line is the non-parametric state occupation probability for the validation data and brown area is the corresponding 95% confidence interval derived from a bootstrap ($N_{bootstrap} = 1000$).
